# The Efficacy of Focused Extracorporeal Shock Wave Therapy and Ultrasound Therapy in the Treatment of Calcar Calcanei: A Randomized Study

**DOI:** 10.1155/2023/8855687

**Published:** 2023-02-20

**Authors:** Ivana Topalović, Dejan Nešić, Sindi Mitrović, Vera Miler Jerković, Ljubica Konstantinović

**Affiliations:** ^1^Faculty of Medicine, University of Belgrade, Serbia; ^2^Faculty of Medicine, University of Belgrade, Institute of Physiology, Serbia; ^3^Faculty of Medicine University of Belgrade, Clinic for Rehabilitation dr Miroslav Zotović, Belgrade, Serbia; ^4^University of Belgrade, Innovation Center of the School of Electrical Engineering, Belgrade, Serbia

## Abstract

The prospective, simple randomized study assesses the effect of focused extracorporeal shock wave therapy (f-ESWT) on pain intensity and calcification size compared to the application of ultrasound physical therapy in treating patients with calcar calcanei. A total of 124 patients diagnosed with calcar calcanei were consecutively included in the study. The patients were divided into two groups: the experimental group (*n* = 62), which included the patients treated with f-ECWT, and the control group (*n* = 62), consisting of patients treated with the standard ultrasound therapy method. The experimental group's patients received ten therapy applications spaced seven days apart. The patients in the control group had ten ultrasound treatments on ten consecutive days over two weeks. All patients in both groups were tested using the Visual Analog Scale (VAS) to measure pain intensity before the beginning and at the end of treatment. The size of the calcification was assessed in all patients. The study hypothesizes that f-ESWT reduces the pain and the size of the calcification. Pain intensity reduction was registered in all patients. The calcification size in patients in the experimental group was reduced from the initial range of 2 mm–15 mm, to a content of 0.0 mm–6.2 mm. The calcification size in the control group ranged from 1.2 to 7.5 mm, without any change. None of the patients experienced any adverse reactions to the therapy. Patients treated with standard ultrasound therapy did not have a statistically significant reduction in the calcification size. In contrast, the patients in the experimental group treated with f-ESWT showed a substantial decrease in the calcification size.

## 1. Introduction

Calcar calcanei is one of the most frequent causes of foot pain in adults (15–20%) [1]. The main symptom of calcar calcanei is heel pain during weight-bearing activities [2]. According to literature data, this disease occurs more frequently in women than men. Calcification occurs most commonly if the footwear is uncomfortable, if the patient is obese, or due to a preexisting foot deformity [3, 4].

Calcar calcanei is a disease manifested by the presence of calcification on the calcaneus. Histological analysis shows that the calcar calcanei consists of a core of mature lamellar bone and demonstrates evidence of degeneration and fibro-cartilaginous proliferation, along with one or more intramembranous, chondroid, and endochondral ossifications occurring at the surface [5]. Calcification is most often found at the point of attachment of the plantar fascia (PF) to the calcaneus, and it is closely related to plantar fasciitis (PFis). Excessive stresses lead to a chronic degenerative condition of PFis that is histologically characterized by fibroblastic hypertrophy, absence of inflammatory cells, disorganized collagen, and chaotic vascular hyperplasia with zones of avascularity and even ossification at the fascial insertion point on the calcaneus [6, 7]. The lack of elasticity of the affected PF due to degenerative processes could be demonstrated using shear wave elastography (SWE) [8].

Its treatment is multimodal and may include the following: the application of anti-inflammatory drugs, physical therapy, surgical treatment, kinesiotherapy, and the use of an orthosis. Generally speaking, there is no proof of the efficiency of any particular method, apart from glucocorticoid infiltration, which is associated with possible atrophy of the tissue in the foot heel or rupture of the plantar fascia [9].

Nowadays, in the literature, there is numerous evidence of the use of f-ESWT in the treatment of plantar fasciitis without or with (calcar calcanei) calcification. [10–13]. The effects of treatment with f-ESWT, which are exploited in this context, are the indirect effects of increased inflammatory indices, neovascularization, and tissue regeneration. The final effect of this treatment is the return of the biological and functional properties of the tissue.

The effect of the application of ultrasound therapy is twofold. The thermal effect manifests as a local increase in the temperature of the tissue located at the site of therapy application. The mechanical effect leads to micro-massage of the tissue being treated, resulting in increased permeability of cell membranes. This therapy has the effect of reducing swelling and tension in soft tissues, reducing pain and inflammation, and improving sensation.

Research on the efficiency of safe physical therapy methods is an important current issue. Before introducing shock wave therapy into clinical practice, different forms of therapy were applied in treating calcifications. The application of different forms of therapy used thus far, including the local administration of corticosteroids, results in reduced swelling of the soft tissue of the heel and sole of the foot and reduced pain. Surgical removal of the calcification may lead to side effects in the soft tissue of the heel [14]. f-ESWT therapy results in the reduction or complete disappearance of the calcification, while the level of the patient's discomfort experienced during application is the lowest compared to other treatment options [15].

Standard ultrasound therapy in calcar calcanei was the treatment of choice before introducing ESWT [16]. When ultrasound and shock wave therapy are compared, the latter has the advantage, as it improves the quality of life and brings about the breaking up of the calcification, in addition to reducing pain [17].

The study aims to determine the effect of f-ESWT on pain intensity and the calcification size in calcar calcanei compared to the application of standard ultrasound therapy.

## 2. Methods

The protocol applied in this study is based on the principles of the Helsinki Declaration. The study was approved by the Faculty of Medicine Ethics Committee of Belgrade University (No. 213). All of the patients involved in the study submitted written consent forms for inclusion in the study. The research was performed at the Clinic for Rehabilitation dr Miroslav Zotovic. The study was carried out between September 1, 2021, and September 31, 2022.

The study included 124 consecutive patients. With the method of equal randomization, the patients were divided into two groups. The first group was designated as the experimental group and had 62 patients treated with f-ESWT. The second group was selected as the control group; it included 62 patients treated with standard ultrasound therapy.

## 3. Criteria

A total of 190 patients were considered for the study, of whom 124 fulfilled the criteria for inclusion. The primary criterion for including patients in the study was diagnostically confirmed painful calcar calcanei diagnosed with X-rays up to six months before. Patients with the following contraindications were excluded from the study: coagulation disorder, anticoagulant use, carcinoma, pregnancy, sensory polyneuropathy, osteoporosis, a pacemaker, acute conditions, lesions of the sole skin, and cortisone treatment up to six weeks before the initial treatment session. Statistical analysis was performed on an “intention to treat” basis, and dropout was not registered.

## 4. The Procedure

The shock wave therapy machine used was the Masterpuls MP 200, serial No: BS 2058, manufactured in 2011 by STORZ MEDICAL, Switzerland. Shock wave therapy was applied locally, on the heel of the foot, at the site of the calcar calcanei. The application was performed with a focused probe with the following parameters: 16 Hz frequency, 1,600 shocks, and a pressure of 1.6 bars. The patients lay prone, and a cylinder was placed under their lower leg so the heel would be completely relaxed and more accessible to the doctor performing the therapy. The f-ESWT was applied once a week for ten weeks.

The patients in the control group underwent a series of ten physical ultrasound therapy treatments continuously every day. The machine applied was the SONOPULS 492 Enraf-Nonius. The application of standard ultrasound therapy was performed using the stable method, directly on the heel of the foot and the plantar fascia, for five minutes at an intensity ranging between 0.5 and 0.8 W/cm2. The patients were positioned in the same way as for shock wave therapy application. A licensed physician administered shock wave therapy. A licensed physical therapist administered standard ultrasound treatment to the patients in the control group according to the protocol defined by a physiatrist. None of the patients treated in either of the groups took any medication belonging to the group of analgesics or non-steroid antirheumatics, nor were they treated with corticosteroid drugs during the study. Additionally, none used an orthosis for their heel or applied any gels locally.

## 5. Outcome Parameters

The pain intensity was measured using the Visual Analog Scale (VAS) [18]. All patients in both groups were tested with the VAS scale to measure pain at the beginning and end of treatment. The Visual Analog Scale (VAS) is based on the analysis of pain when the patient feels no pain and is experiencing significant pain. As a diagnostic method, this scale has excellent potential for pain assessment and is the most commonly used one-dimensional pain rating scale in clinical practice. The enumeration of the scale enables the numerical quantification of pain intensity, ranging from 0 to 10. The VAS scale can be used repeatedly in the same patient to follow up on the pain evaluation during the treatment. In this study, the VAS scale was used for each patient, with a range of 0 to 10, whereby 10 represented the highest pain intensity and 0 represented no pain. The patients were tested with the VAS scale twice, at the beginning of the course of treatment at the end.

The calcification size was measured in millimeters with X-ray diagnostics. Imaging the calcar calcanei was performed by applying the standard X-ray technique for the ankle, with the patient standing upright and the X-ray beam spread vertically. Thus, the images show all the elements of the ankle (distal tibia, talus, and calcaneus). The size and angle of each abnormality of the calcaneus, which was registered as a calcar calcanei, were measured. The measurement of the size of the calcar calcanei was performed via the calcaneal inclination angle (CIA), the lateral talocalcaneal angle (LTCA), the Böhler angle (BA), and the Gissane angle [19, 20].

All the results were statistically processed with the IBM SPSS Statistics 22 (SPSS Inc., Chicago, IL, USA) software package.

### 5.1. Statistical Analysis

Descriptive statistics were used to describe data. The continuous variables are presented by mean ± standard deviation, while the categorical variables are presented by count (percentage).

The comparison between independent variables, which distributions were approximately the same as a normal distribution, we were done by the *t*-test for independent data in the case of equal variance or by the Welch *t*-test in the case of unequal variances. The Mann–Whitney test compares independent variables, which distributions are not the same as a normal distribution. The comparison between dependent variables, which distributions were approximately the same as a normal distribution, we were done by the t-test for dependent data. The comparison between dependent variables, which distributions were not the same as a normal distribution, we were done by the Wilcoxon test. The testing of the normality of the distribution of each variable was done by using descriptive statistics, Kolmogorov–Smirnov, and Shapiro–Wilks and graphical methods. The Cohen effect size [21] was calculated for any test. According to Cohen [16], the interpretation for a small, moderate, and large Cohen effect size is *d* = 0.20, 0.50, and 0.80. The Cliff's *d* effect size [22] was used for the Mann–Whitney test. According to Cliff [23], this effect size is appropriate for comparing two groups. The interpretation of Cliff's d for small, medium, and large is 0.15, 0.33, and 0.48. The rank biserial correlation coefficient rs [22] was used as the effect size for the Wilcoxon test. The interpretation for the effect size for small, medium, and large is 0.10, 0.30, and 0.50. The categorical variables were compared by using the Chi-Squared test. The Cramér's *V* [24] effect size was calculated for the Chi-Squared test. The interpretation for Cramer V effect size for small, medium, and large is 0.10, 0.30, and 0.50.

The relationship between variables for each group was analyzed separately by Pearson's correlations. The relationship between the two variables was analyzed by controlling the third variable using partial correlation. The partial correlation coefficient measures the strength of the linear relationship between two variables after entirely controlling for the effects of other variables. The logistic regression was performed for changes in a specific outcome.

The level of statistical significance was set at a two-tailed alpha level of 0.05. RStudio (version 1.4.1106) was used for statistical analysis.

### 5.2. Results

**Figure 1:**
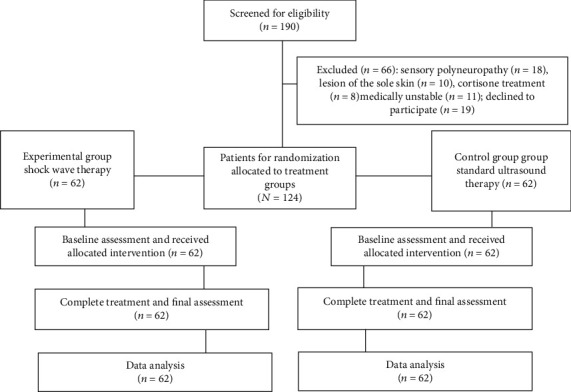
Flow diagram of patient recruitment.

One hundred and ninety consecutive patients with diagnostically confirmed painful calcar calcanei were screened for eligibility criteria. One hundred and twenty-four patients fulfilled all eligibility criteria. Eighteen patients had sensory polyneuropathies, a lesion of the sole skin had ten participants, cortisone treatment up to six weeks before the initial treatment session had eight, eleven patients were medically unstable, and nineteen declined to participate in the clinical trial. The patients were divided by the method of equal randomization into two groups. The first group was designated as the experimental group and had 62 patients treated with shock wave therapy. The second group was selected as the control group; it included 62 patients treated with standard ultrasound therapy.  Figure 1 shows a flow diagram of patient recruitment throughout the study.

Descriptive statistics of all outcomes for both groups are presented in Table 1.

The results of the analysis of comparisons between experimental and control groups are presented in Table 2. The statistically significant differences in baseline outcomes between the experimental group and control group were found at therapy duration (*p* = 0.005) with large effect size and calcification posttherapy (*p* < 0.0001) and medium effect size.

The results of the ‘pre-post' therapy analysis are presented in Table 3. The statistically significant differences were obtained in both groups at parameters calcification and VAS with large effect sizes.

Calcification and VAS change were calculated as subtraction, pretherapy, posttherapy, and VAS pretherapy and VAS posttherapy. The results of comparing changes in calcification and VAS, separately between the experimental and control groups are presented in Table 4. A statistically significant difference was obtained at calcification between subjects from the experimental and control group (*p* < 0.0001) with a large effect size.

Additionally, we observed the change in the VAS as greatly improved (> = 50), much improved (50 < ×< = 30), somewhat improved (30 < ×< = 10), about the same (10 < ×< = 1), and worse (>1). The change in VAS in both groups is presented in Table 5. The result (*χ*2 = 2.37, *p* = 0.864) was shown that there is no statistically significant difference between VAS improvement between experimental and control groups.

Additionally, we observed the change in the calcification as improved (>0), not improved (<=0)—the change of calcification in both groups presented in Table 6. The result (*χ*2 = 15.34, *p* = 0.0005) showed a statistically significant difference in calcification improvement between experimental and control groups.

The logistic regression was done for calcification improvement, and the results are presented in Table 7. The odds for subjects in the experimental group to have calcification improvement are 21.2 higher than for subjects in the control group.

The relationship between variables for each group was analyzed separately by Pearson's correlations. The results are shown in Table 8. The significant correlations (*p* < 0.05) were marked in bold italic. In the experimental group, the positive correlation between calcification change and age is moderate (*r* = 0.36, *p* = 0.004) and shows that improvement in calcification after therapy is more significant for older subjects; the positive correlation between calcification change and BMI is moderate (*r* = 0.31, *p* = 0.01) and shows that improvement in calcification after therapy is more significant for subjects with bigger BMI. In the control group: the positive correlation between calcification change and BMI is a moderate degree (*r* = 0.38, *p* = 0.002) and shows that improvement in calcification after therapy is more significant for subjects with bigger BMI; the negative correlation between VAS change and BMI is a low degree (*r* = −0.26, *p* = 0.04) and shows that improvement in VAS after therapy is more significant for subjects with lower BMI.

The analysis of the relationship between two variables, calcification changes and BMI, while controlling by the third variable, age, was done using the partial correlation. The partial correlation was statistically significant and positive with a small degree (*r* = 0.26, *p* = 0.01). The controlling variable, age, had little effect on the relationship between calcification changes and BMI.

The analysis of the relationship between two variables, calcification changes and age, while controlled by the third variable, BMI, was done using the partial correlation. The partial correlation was statistically significant and positive with a medium degree (*r* = 0.32, *p* = 0.01). The controlling variable, BMI, had little effect on the relationship between calcification changes and age.

The analysis of the relationship between two variables, calcification change and VAS change, but controlling a third variable (age, BMI, and therapy duration, separately) was done using the partial correlation. The partial correlation coefficient measures the strength of the linear relationship between two variables after entirely controlling for the effects of other variables. The results are presented in Table 9.

## 6. Discussion

The most prominent symptom of calcar calcanei is pain. In our study, none of the patients from either of the groups took any analgesics or nonsteroid antirheumatics. Analyzing the results obtained in treating the patients in the experimental and control groups, we received statistically similar data regarding the decrease in pain levels in patients. The application of f-ECWT in the experimental group of patients showed a demonstrable reduction in the calcification size and a decrease in pain. Patients from the control group treated with standard ultrasound therapy experienced a positive effect regarding pain intensity reduction. By analyzing the calcification size in millimeters, in patients of both tested groups, a result was obtained demonstrating the effect of f-ESWT on reducing the calcification. Correlation analyses did not show a connection between the size of calcification and pain, but some factors, such as BMI and the age of patients, moderate the therapeutic effects. The results show that the improvement in calcification size is more significant for the elderly and subjects with a higher BMI. There is no data in the literature on the moderating effect of other factors related to patient characteristics. Side effects of the application of shock wave therapy were not registered. Similarly, there were no registered side effects among the patients in the control group.

Many studies confirm that the f-ESWT method is noninvasive, safe, and without severe contraindications for breaking up calcifications [25–27]. The experience of various authors is that they used f-ESWT at an intensity ranging between 10 and 21 bars, a frequency of 20 Hz, and some shocks ranging from two to four thousand [28]. The suggestions regarding the frequency of f-ESWT application, i.e., the length of time that should elapse between two applications of shock wave therapy, vary in the literature.

The complete biological mechanism of shock wave therapy has yet to be precisely understood. It is believed that ESWT promotes healing through the process of mechano-transduction, acting as a mechanical stimulus [29, 30]. The studies have suggested that mechano-transduction is the central mechanism whereby ESWT triggers angiogenic and bone remodeling responses at cellular and molecular levels, generating beneficial therapeutic effects such as pain relief and tissue regeneration by stimulating vascularization and reducing calcium deposits in tissues [31]. Additionally, it may also alleviate pain through hyperstimulation analgesia [32]. Applying ESWT stimulates the proliferation, migration, and differentiation of stem cells, fibroblasts, tenocytes, bone cells, and their precursors [33–35]. To understand the effects shown in this study, studies that show the direct influence of periosteal stimulation on orthotopic bone regeneration and the reduction of osteoclast activity through the inhibition of pro-osteoclastogenic factors could be considered. [36]. The research results on tenocyte activity indicate increased production of collagen and decreased expression of metalloproteinases and inflammatory cytokines, essential in remodeling plantar fasciitis [37, 38]. Some of the biological responses on a molecular level include increased early expression of vascular endothelial growth factor (VEGF), endothelial nitric oxide synthase (eNOS), and proliferating cell nuclear antigen (PCNA), which leads to neovascularization and better blood supply. [39, 40]. At the cellular level, it has been shown that ESW can induce transient deformation of some cytoskeletal proteins (actin and tubulin, but not vimentin), but reorganization into their original cytoskeletal network appeared within 3 hours, with a pattern similar to the control [41]. However, the primary biological effect of ESWT is stimulative. In recent years, the interest of researchers in studying the regenerative effect of shock wave therapy has increased [31].

It is assumed that the application of f-ESWT induced changes at the cellular and molecular level, resulting in a reduction in the calcification size. An objective reduction in calcification size was also statistically proven in this study.

## 7. Study Limitations

The result should be considered against a few limitations. The patients are not homogeneous in the duration of symptoms and have not been monitored for a long time. A limitation is the lack of determination of the sample size since the patients were determined consecutively. Also, many factors other than BMI that could influence the effects of therapy were not monitored.

## 8. Conclusion

In conclusion, both types of therapy applied in the study contributed to reducing pain intensity. The application of f-ESWT significantly reduced the calcification size compared with standard ultrasound therapy.

## Figures and Tables

**Table 1:** Descriptive statistics of all outcomes in this study.

Parameters	Experimental group	Control group	*p* value
Age	54.48 ± 9.86	51.92 ± 14.35	0.249
BMI	29.25 ± 2.48	28.50 ± 2.31	0.111
Therapy duration	2.82 ± 0.84	2.37 ± 1.37	***0.005***
Calcification pretherapy	4.21 ± 1.39	4.27 ± 1.17	0.605
Calcification posttherapy	2.25 ± 0.98	3.33 ± 1.01	***8.52e-08***
VAS pretherapy	76.23 ± 8.75	74.73 ± 11.35	0.773
VAS posttherapy	36.98 ± 8.04	39.35 ± 8.15	0.083
Leg			
Right	40 (64.5%)	0.07	
Left	22 (35.5%)	32 (51.6%)	
Sex			
Male	29 (46.8%)	0.59	
Female	33 (53.2%)	30 (48.4%)	

**Table 2:** The comparisons of outcomes with descriptive statistics (mean ± standard deviation), test statistics, *p* values and effects size.

Parameters	Experimental group	Control group	Statistics	*p* value	Effect size
Age	54.48 ± 9.86	51.92 ± 14.35	1.16	0.249	—
BMI	29.25 ± 2.48	28.50 ± 2.31	2241	0.111	—
Therapy duration	2.82 ± 0.84	2.37 ± 1.37	2468	***0.005***	0.65^C^
Calcification size pretherapy	4.21 ± 1.39	4.27 ± 1.17	1818	0.605	—
Calcification size posttherapy	2.25 ± 0.98	3.33 ± 1.01	851	***8.52e-08***	0.23^C^
VAS pretherapy	76.23 ± 8.75	74.73 ± 11.35	1980	0.773	—
VAS posttherapy	36.98 ± 8.04	39.35 ± 8.15	1575	0.083	—
Leg					
Right	40 (64.5%)	30 (48.4%)	3.28	0.07	—
Left	22 (35.5%)	32 (51.6%)			
Sex					
Male	29 (46.8%)	32 (51.6%)	0.29	0.59	—
Female	33 (53.2%)	30 (48.4%)			

^d^the Cohen effect size; ^C^the Cliff s effect size, ^K^the Cramér's *V* effect size.

**Table 3:** The ‘pre-post' analysis of VAS and calcification.

Group	Parameters	Pretherapy	Posttherapy	Statistics	*p* value	Effect size
Experimental	Calcification	4.21 ± 1.39	2.25 ± 0.98	1891	***<0.0001***	0.79^rs^
	VAS	76.23 ± 8.75	36.98 ± 8.04	1953	***<0.0001***	0.89^rs^
Control	Calcification	4.27 ± 1.17	3.33 ± 1.01	1102	***<0.0001***	0.48^rs^
	VAS	74.73 ± 11.35	39.35 ± 8.15	1891	***<0.0001***	0.80^rs^

^d^the Cohen effect size: ^rs^the rank biserial correlation coefficient.

**Table 4:** The analysis of change of calcification and VAS.

Change	Experimental group	Control group	Statistics	*p* value	Effect size
Calcification	1.96 ± 1.04	0.94 ± 1.1	2959	***<0.0001***	0.54^C^
VAS	39.24 ± 10.01	35.37 ± 12.35	1.92	0.058	0.34^d^

^d^the Cohen effect size; ^C^the Cliff s effect size.

**Table 5:** The distribution of VAS change.

VAS change	Experimental group	Control group
Greatly improved	9 (14.5%)	9 (14.5%)
Much improved	39 (62.9%)	40 (64.5%)
Somewhat improved	14 (22.6%)	11 (17.7%)
About the same	0 (0%)	1 (1.6%)
Worse	0 (0%)	1 (1.6%)

**Table 6:** The distribution of calcification change.

Calcification	Experimental group	Control group
Improved	61 (98.4%)	46 (74.2%)
Not improved	1 (1.16%)	16 (25.8%)

**Table 7:** The logistic regression.

Coefficients	Estimate	Wald *z* statistics	*p* value	Odds
Intercept	1.06	3.64	0.0003	2.87
Group	3.06	2.91	***0.0004***	21.2

**Table 8:** The correlation coefficients for both groups, separately.

Group	Age	BMI	Calcification change
Experimental			
Age			
Bmi	0.21		
Therapy duration	0.15	−0.15	
Calcification change	***0.36***	***0.31***	
VAS change	−0.24	−0.18	−0.08
Control			
Age			
Bmi	0.12		
Therapy duration	0.06	−0.06	
Calcification change	0.11	***0.38***	
VAS change	−0.02	***−0.26***	−0.03

**Table 9:** The partial correlation analysis.

Group	Relationship	Age	BMI
Experimental	Calcification change~VAS change	0.001	−0.03
Control	Calcification change~VAS change	−0.03	0.07

## Data Availability

Data supporting the study results can be found in the medical data system of the Clinic for Rehabilitation dr Miroslav Zotovic.
